# Differential association of dietary scores with the risk of type 2 diabetes by metabotype

**DOI:** 10.1007/s00394-024-03411-0

**Published:** 2024-05-07

**Authors:** Zhongyi Deng, Nina Wawro, Dennis Freuer, Annette Peters, Margit Heier, Christine Meisinger, Taylor A. Breuninger, Jakob Linseisen

**Affiliations:** 1 Institute for Medical Information Processing, Biometry, and Epidemiology – IBE, Ludwig- Maximilians University of Munich, Marchioninistr. 15, 81377 Munich, Germany; 2 Pettenkofer School of Public Health, Ludwig-Maximilians University of Munich, Pettenkoferstr. 9A, 80336 Munich, Germany; 3 Chair of Epidemiology, Medical Faculty, University of Augsburg, University Hospital of Augsburg, Stenglinstr. 2, 86156 Augsburg, Germany; 4 Institute of Epidemiology, Helmholtz Munich (GmbH) – German Research Center for Environmental Health, Ingolstädter Landstr. 1, 85764 Neuherberg, Germany; 5 German Center for Diabetes Research (DZD), Munich-Neuherberg, Germany; 6 KORA Study Centre, University Hospital Augsburg, Beim Glaspalast 1, 86153 Augsburg, Germany

**Keywords:** Type 2 diabetes, Glucose tolerance status, Dietary scores, Metabotype, Ultra-processed foods, Nutri-score

## Abstract

**Purpose:**

We aimed to examine the association between dietary patterns and type 2 diabetes mellitus (T2DM) while considering the potential effect modification by metabolic phenotypes (metabotypes). Additionally, we aimed to explore the association between dietary scores and prediabetes.

**Methods:**

A total of 1460 participants (11.8% with T2DM) from the cross-sectional population-based KORA FF4 study were included. Participants, classified into three metabotype subgroups, had both their FSAm-NPS dietary index (underpinning the Nutri-Score) and ultra-processed foods (UPF) intake (using NOVA classification) calculated. Glucose tolerance status was assessed via oral glucose tolerance tests (OGTT) in non-diabetic participants and was classified according to the American Diabetes Association criteria. Logistic regression models were used for both the overall and metabotype-stratified analyses of dietary scores’ association with T2DM, and multinomial probit models for their association with prediabetes.

**Results:**

Participants who had a diet with a higher FSAm-NPS dietary index (i.e., a lower diet quality) or a greater percentage of UPF consumption showed a positive association with T2DM. Stratified analyses demonstrated a strengthened association between UPF consumption and T2DM specifically in the metabolically most unfavorable metabotype (Odds Ratio, OR 1.92; 95% Confidence Interval, CI 1.35, 2.73). A diet with a higher FSAm-NPS dietary index was also positively associated with prediabetes (OR 1.19; 95% CI 1.04, 1.35).

**Conclusion:**

Our study suggests different associations between poorer diet quality and T2DM across individuals exhibiting diverse metabotypes, pointing to the option for stratified dietary interventions in diabetes prevention.

**Supplementary Information:**

The online version contains supplementary material available at 10.1007/s00394-024-03411-0.

## Introduction

Type 2 diabetes mellitus (T2DM) is a chronic metabolic disorder that affects millions of people worldwide and poses a substantial burden to human health and economy [1, 2]. The global prevalence of diabetes in 2021 was estimated to be 10.5% (537 million people), projected to increase to 12.2% (783 million people) by 2045 [3]. T2DM is associated with various comorbidities, including cardiovascular disease, neuropathy, retinopathy, and kidney disease, leading to significant morbidity and mortality [4]. Prediabetes is a high-risk state for diabetes defined as blood glucose levels above normal but below diabetes thresholds [5]; up to 10% of individuals with prediabetes may develop T2DM annually [6]. Existing metabolic abnormalities during the prediabetes stage can heighten the risk of multiple comorbidities and chronic complications typically associated with diabetes [3, 6], underscoring the urgency for early intervention strategies.

Many risk factors contribute to diabetes development, including genetics and modifiable lifestyle factors such as diet, smoking, and physical activity [2]. Among them, diet plays a crucial role and some food groups or nutrients have already been linked to the T2DM [1, 2]. Given the complexity of individuals’ eating habits, dietary patterns that describe the overall diet quality may better reflect the diet’s impact on multifactorial diseases than isolated components.

In the past, many dietary indices were designed to evaluate different characteristics or patterns of habitual food consumption. More recently, dietary scores based on the Nutri-Score and the NOVA classification system [7, 8] have become available. The Nutri-Score is a front-of-pack food labeling system computed from the Modified Food Standards Agency Nutrient Profiling System (FSAm-NPS), grading the overall nutritional quality of the food [7]. On the other hand, the NOVA classification rates foods based on their degree of processing, rather than solely on nutrient content [8]. Ultra-processed foods (UPF) belong to the fourth group of the NOVA classification and have undergone the highest degree of industrial processing, potentially impacting health via multiple mechanisms [8–10]. Both dietary indices have been widely applied to assess diet quality and ultimately may help to reduce the diet-related burden of non-communicable diseases in society [11–13]. Despite several investigations demonstrating a consistent association between UPF consumption and T2DM [14, 15], as well as the high Nutri-Score diet with elevated blood glucose [16, 17], the influence of individuals’ metabolic characteristics on these linkages remains largely unexplored.

Studies suggest that personalized and metabolism-specific dietary recommendations may surpass general advice in terms of their efficacy in improving eating behavior and influencing health outcomes [18–21]. To address this, one possible solution is to stratify the population into subgroups, termed metabotypes [18], according to similarities in their metabolic profiles and develop tailored preventive measures. Building on our previous work identifying various diet-T2DM associations across metabotypes [22, 23], we aimed to investigate how metabotype subgrouping may affect the dietary score and T2DM association and the implications for disease prevention. We hypothesize that strata-specific dietary interventions could be beneficial. Additionally, we sought to explore the association between dietary patterns and prediabetes. The analysis focused on the FSAm-NPS dietary index (underpinning the Nutri-Score) and UPF intake ratio (using NOVA classification).

## Methods

This study’s findings were reported by following the “Strengthening the Reporting of Observational Studies in Epidemiology-Nutritional Epidemiology (STROBE-nut)” checklist [24].

### Population

Our analysis was based on data from the German Cooperative Health Research in the Augsburg region (KORA) FF4 study (2013–2014, 2279 participants), a follow-up to the F4 (2006–2008, 3080 participants) and S4 baseline study (1999–2001, 4261 participants), conducted in a randomly selected general population in Augsburg and two surrounding counties. Study designs have been described in detail elsewhere [25]. Out of 2279 FF4 participants, subjects with missing dietary information (n = 677), type 1 diabetes (n = 3), unclear glucose tolerance status due to missing oral glucose tolerance test (OGTT) information (n = 39), or missing metabotype information (n = 24) were excluded. Participants with a diagnosis of myocardial infarction (n = 43) or stroke (n = 31) were further excluded as severe disease might change dietary behavior. A flowchart of exclusion criteria is shown in Supplementary Fig. 1. A total of 1460 participants were eventually selected for our cross-sectional analysis.

The KORA study was authorized by the Ethics Committee of the Bavarian Medical Society and conducted in accordance with the Helsinki Declaration. All study subjects provided written informed consent.

### Dietary assessment and dietary indices

The participants’ usual dietary intake was assessed by means of one food frequency questionnaire (FFQ) and repeated 24-hour food lists (24HFL). During the study center visit, participants were required to complete a first 24HFL that assesses foods that were consumed over the previous day; up to two further 24HFLs were collected during the next three months. The 24HFL comprised 246 food items and was derived from the German National Cohort (NAKO) Health study [26]. The FFQ, adapted from the German version of the multilingual European Food Propensity Questionnaire [27], queries the consumption frequency of 148 food items over the past 12 months. Each participant’s usual daily food intake was determined by combining estimated consumption probability and amount, and the detailed has been described elsewhere [28]. The validity of this approach is supported by evidence showing that the combined use of multiple 24-hour recalls and FFQ data provide more accurate intake estimates as compared to either method applied alone [29]. In addition, the usual intake estimates of food items were categorized into main food groups and subgroups based on the EPIC-Soft classification system [30] and estimates of nutrient intake were derived for every participant by linking the usual intake estimates to the German Nutrient Database (Bundeslebensmittelschlüssel 3.02).

In order to indicate participants’ overall dietary quality, we calculated the FSAm-NPS dietary index [7] and the proportion of UPF intake [8] based on their daily consumption estimates.

The FSAm-NPS dietary index assesses the overall nutritional quality of an individual’s diet based on the nutrient profiling system (NPS). It is slightly adjusted to the allocation of points for specific foods (beverages, cheese, and added fats) recommended by the French High Council of Public Health as a modified version of the original Nutrient Analysis System (FSA-NPS) [7, 31]. This ensures that the FSAm-NPS score corresponds to nutritional recommendations and that the nutritional quality of products within these groups can be distinguished [31]. The FSAm-NPS score’s calculation method is described in more detail elsewhere [7, 31, 32]. Theoretically the FSAm-NPS score falls on a scale from − 15 (the healthiest option) to 40. In practice, the Nutri-Score labeling system classifies foods and beverages into five categories, from category A (indicating higher nutritional quality) to category E (indicating lower nutritional quality), based on the FSAm-NPS score [7]. We calculated the FSAm-NPS dietary index for each participant’s diet by adding the FSAm-NPS score for each food or beverage consumed, multiplying it by the amount of energy provided by this product (energy content per 100 g multiplied by the estimated daily intake assessed), and dividing it by the total amount of energy intake [33]. Increasing values of the FSAm-NPS dietary index thus indicate poorer overall diet quality.

UPF intake was estimated using the NOVA classification [8], which categorizes foods based on the level and intensity of industrial processing: (1) fresh/minimally processed foods (e.g., fruit, meat, milk); (2) processed culinary ingredients (e.g., oils, butter, sugar); (3) processed foods (e.g., canned fish, fresh unpackaged bread); and (4) ultra-processed foods (UPFs), made mostly or entirely from derived food constituents with added flavors, colors, and other additives (e.g., sugar-sweetened drinks, processed meat, and savory packaged snacks). To determine the proportion of UPF consumption of the total dietary intake for each participant, we summed the quantities (in kcal/d and gram/d) of each food group from all four categories of NOVA for the total diet and calculated the proportion (%) of UPF (in kcal/d and gram/d, respectively) of the total diet, which is called energy ratio (or weight ratio) in our study. Individuals with a higher UPF intake ratio tend to have a poorer diet quality.

### Metabotypes

Aiming at metabolic homogeneity within groups, our study population was divided into three clusters (metabotypes). Further details are described elsewhere [34]. The metabotyping process used 5 parameters, i.e., fasting glucose, high density lipoprotein (HDL) cholesterol, non-HDL cholesterol, uric acid, and body mass index (BMI), to identify clusters by applying the k-means clustering algorithm. Here, we used the Euclidean distances of the three cluster centers as determined in KORA F4 to allocate the KORA FF4 participants to three metabolic clusters [34], using the parameter values measured in fasting blood samples collected during the FF4 study center visit. Among the three clusters, cluster 1 is deemed the most metabolically favorable (“healthy metabotype”), cluster 3 the least favorable, and cluster 2 an “intermediate metabotype” between the other two [34].

### Measurement of covariates

Information such as age, sex, education, hypertensive status, alcohol consumption, fasting status, smoking status, and physical activity was assessed by trained medical staff through a standardized interview. The classification of the education variable was determined according to the educational system in Germany. Waist circumference was measured midway between the lowest margin of the least palpable rib and the top of the iliac crest using stretch-resistant tapes. Sitting blood pressure was measured by a trained health worker using an electronic sphygmomanometer. Measurements were conducted after a rest of at least 5 min on the right arm three times. For the analysis, the second and third measurements were averaged.

Blood samples were collected after an overnight fast of ≥ 8 h and at 2 h after ingestion of a 75 g glucose solution in the absence of stasis and stored at 4 °C until centrifugation. Serum glucose was measured with hexokinase/glucose-6-phosphat-dehydrogenase (GLUFlex; Dade Behring, USA). Triglycerides was enzymatically measured in serum (glycerine phosphate oxidase peroxidase method) (TGL Flex, Dade Behring). HDL cholesterol, low density lipoprotein (LDL) cholesterol and total cholesterol were measured in serum by enzymatic methods (CHOL Flex and AHDL Flex, Dade Behring). High-sensitivity C-reactive protein (hs C-reactive protein) was determined by nephelometry on a BN II analyzer (Siemens, Erlangen, Germany) from frozen plasma. Serum concentrations of uric acid were measured from fresh samples by the uricase method (enzymatic color test, URCA Flex®; Dade Behring). More technical details on the processing of blood samples and biomarker measurements could be found elsewhere [35, 36].

BMI was calculated as weight (kg) per height (m^2^) and categorized into underweight: BMI < 18.5 kg/m^2^; normal weight: 18.5 ≤ BMI < 25 kg/m^2^; overweight: 25 ≤ BMI < 30 kg/m^2^; obese: BMI ≥ 30 kg/m^2^ [37]. Hypertension was defined as current hypertension (≥ 140/90 mmHg) and/or known hypertension controlled by medication. Participants were categorized as “physically active” if they spent ≥ 1 h per week on leisure physical activity in at least one season (summer or winter); otherwise, they were “physically inactive” [38].

### Glucose tolerance status

Glucose tolerance status was categorized into normal glucose tolerance, prediabetes, and T2DM, following the American Diabetes Association criteria [39]. Normal glucose tolerance was defined as fasting glucose < 5.6 mmol/L or 2-h OGTT < 7.8 mmol/L. Prediabetes was defined based on the presence of impaired glucose tolerance (2-h OGTT concentration of 7.8–11.0 mmol/L), impaired fasting glucose (fasting glucose concentration of 5.6–6.9 mmol/L), or both. Undetected diabetes was defined as fasting glucose ≥ 7.0 mmol/L or 2-h OGTT ≥ 11.1 mmol/L in individuals without prevalent, i.e. already known, diabetes. While prevalent diabetes referred to a known diagnosis of T2DM or use of anti-diabetic medication, verified by consulting their treating physician. The OGTT test was conducted in all participants without a previous physician-confirmed diagnosis of T2DM after an overnight fasting period of at least 8 h. We classified individuals as having T2DM if they fell into either the undetected or the prevalent diabetes category.

### Statistical analysis

We analyzed a sample of 1460 individuals from the KORA FF4 study. Baseline characteristics were reported stratified by the tertiles of UPF intake (energy ratio). Daily intakes of major food groups and macronutrients were presented separately stratified by tertiles of dietary scores. Normally distributed continuous covariates were reported as means (with standard deviation (SD)), non-normally distributed continuous covariates as medians with the interquartile ranges. Categorical variables were presented as absolute frequencies and proportions in the form of percentages. We compared differences between groups by using the Chi-square test or Fisher’s exact test for categorical variables and analysis of variance (ANOVA) test or Kruskal-Wallis test for continuous variables.

We used multivariable logistic regression models to study the association between dietary scores and T2DM. The ratio of UPF intake was calculated per 5% increase in UPF intake, and the FSAm-NPS dietary index was scaled per 2-point increase in FSAm-NPS points. These models were constructed based on potential confounders according to previous literature. Model 1 was adjusted for age, sex, and total energy intake, while Model 2 included additional adjustments for education, physical activity, and smoking. To avoid overfitting, total energy intake was excluded in models concerning UPF intake energy ratio. Furthermore, we adjusted for metabotype to investigate its effect on this relationship in both models. To evaluate the effect modification of metabotype, we conducted likelihood-ratio tests. Due to significant interactions between metabotype and dietary scores, metabotype-stratified analyses were also employed.

As the assumptions of ordinal regression were violated, multinomial probit models were used to assess the association between dietary scores and glucose tolerance status. The choice of a multinomial probit model over a multinomial regression model was motivated by the results of the Hausman-McFadden test, which indicated a violation of the assumption of irrelevant alternatives (IIA) [40]. Glucose tolerance status was categorized into normal, prediabetes, and T2DM. The adjusted variables were consistent with the logistic regression models mentioned above.

In sensitivity analyses, we further adjusted for waist circumference and hypertension status (Model 3) and replaced waist circumference with BMI in Model 3 alternatives considering their potential mediation role [41–43]. To assess the impact of carbohydrates intake on results, we conducted additional analyses (Model 4), adjusting for this factor. We calculated odds ratio (OR) with 95% confidence interval (CI) from the logistic regressions and multinomial probit models. All P values correspond with two-tailed tests. P-values of < 0.05 were considered significant. All statistical analyses were performed using R V.4.1.1.

## Results

Among 1460 adults (52.7% of them being women) aged 38–87 years eligible for the study, 173 (11.8%) had T2DM. The mean UPF energy ratio in participants’ diets was 38% (SD = 7%), accompanied by a weight ratio of 16% (SD = 7%) and an FSAm-NPS dietary index averaging 6.93 points (SD = 1.35). Demographic data and comorbidity prevalence by tertiles of UPF intake energy ratio were summarized in Table 1. Compared with the other two groups, participants in the highest UPF intake group were on average younger and male. They had the highest BMI, the largest waist circumference and thus were more often classified as obese. They also showed higher mean triglyceride and C-reactive protein, and lower HDL cholesterol levels than other groups. Furthermore, the highest prevalence of T2DM was observed in this group. We found that participants belonging to metabotype cluster 3 were, on average, the oldest, most likely to be male, and generally had the most unfavorable lifestyle (Supplementary Table S2).

**Table 1:** Baseline characteristics of KORA FF4 study participants, overall and stratified by tertiles of ultra-processed foods (UPF) intake

		Tertiles UPF intake, energy ratio		
	Overall	Low	Medium	High
N	1460	487	486	487
Sex, n (%)				
Male	690 (47.3)	169 (34.7)	231 (47.5)	290 (59.5)
Female	770 (52.7)	318 (65.3)	255 (52.5)	197 (40.5)
Age (years)	59.2 (11.8)	61.0 (11.0)	60.2 (11.9)	56.3 (12.0)
Education, n (%)				
< 10 years	76 (5.2)	23 (4.7)	31 (6.4)	22 (4.5)
10–12 years	837 (57.3)	235 (48.3)	285 (58.6)	317 (65.1)
≥ 13 years	547 (37.5)	229 (47.0)	170 (35.0)	148 (30.4)
Family history of diabetes, n (%)				
Yes	472 (32.3)	161 (33.1)	145 (29.8)	166 (34.1)
No	856 (58.6)	277 (56.9)	300 (61.7)	279 (57.3)
Not specified	132 (9.0)	49 (10.1)	41 (8.4)	42 (8.6)
Metabotype, n (%)				
1	218 (14.9)	74 (15.2)	65 (13.4)	79 (16.2)
2	1029 (70.5)	365 (74.9)	356 (73.3)	308 (63.2)
3	213 (14.6)	48 (9.9)	65 (13.4)	100 (20.5)
BMI (kg/m^2^)	27.46 (4.84)	26.38 (4.20)	27.42 (4.32)	28.58 (5.61)
BMI categorized, n (%)				
Underweight	6 (0.4)	5 (1.0)	1 (0.2)	0 (0.0)
Normal weight	476 (32.6)	195 (40.0)	153 (31.5)	128 (26.3)
Overweight	611 (41.8)	197 (40.5)	216 (44.4)	198 (40.7)
Obese	367 (25.1)	90 (18.5)	116 (23.9)	161 (33.1)
Waist circumference (cm)	95.8 (14.1)	92.3 (12.7)	95.8 (13.3)	99.3 (15.3)
Physical activity, n (%)				
Active	909 (62.3)	379 (77.8)	299 (61.5)	231 (47.4)
Inactive	551 (37.7)	108 (22.2)	187 (38.5)	256 (52.6)
Smoking status, n (%)				
Current	204 (14.0)	77 (15.8)	62 (12.8)	65 (13.3)
Former	560 (38.4)	177 (36.3)	185 (38.1)	198 (40.7)
Never	696 (47.7)	233 (47.8)	239 (49.2)	224 (46.0)
Alcohol consumption (g/day)	7.0 [0.2, 22.9]	5.7 [0.2, 22.9]	8.6 [1.4, 22.9]	5.7 [0.0, 20.3]
Fasting serum glucose (mg/dl)	97.0 [91.0, 106.0]	97.0 [91.0, 106.0]	97.0 [91.0, 106.0]	98.0 [91.0, 107.0]
hs C-reactive protein (mg/L)	1.12 [0.57, 2.40]	1.04 [0.50, 2.12]	1.13 [0.60, 2.29]	1.25 [0.61, 2.93]
Total-cholesterol (mg/dl)	218.8 (39.5)	221.5 (38.5)	218.7 (39.3)	216.3 (40.6)
HDL cholesterol (mg/dl)	64.0 [53.0, 78.6]	69.6 [57.3, 84.0]	65.0 [53.9, 79.7]	60.6 [50.0, 72.0]
non-HDL cholesterol (mg/dl)	152.0 (40.1)	150.8 (40.0)	150.9 (38.8)	154.3 (41.3)
LDL cholesterol (mg/dl)	136.1 (35.5)	135.8 (35.2)	136.0 (35.5)	136.45 (35.8)
Triglycerides (mg/dl)	104.9 [75.7, 142.0]	97.5 [72.0, 134.9]	100.2 [74.0, 137.4]	113.9 [80.6, 157.2]
Serum uric acid (mg/dl)	5.49 [4.51, 6.63]	5.17 [4.39, 6.36]	5.53 [4.55, 6.67]	5.74 [4.66, 6.84]
Hypertension, n (%)				
No	897 (61.5)	307 (63.3)	290 (59.7)	300 (61.6)
Yes	561 (38.5)	178 (36.7)	196 (40.3)	187 (38.4)
Glucose tolerance status, n (%)				
Normal glucose tolerance	771 (52.8)	267 (54.8)	258 (53.1)	246 (50.5)
Prediabetes	516 (35.3)	174 (35.7)	172 (35.4)	170 (34.9)
Undetected diabetes	58 (4.0)	14 (2.9)	21 (4.3)	23 (4.7)
Prevalent diabetes	115 (7.9)	32 (6.6)	35 (7.2)	48 (9.9)
T2DM, n (%)				
No	1287 (88.2)	441 (90.6)	430 (88.5)	416 (85.4)
Yes	173 (11.8)	46 (9.4)	56 (11.5)	71 (14.6)
UPF intake, energy ratio	0.38 (0.07)	0.32 (0.03)	0.38 (0.02)	0.46 (0.04)
UPF intake, weight ratio	0.16 (0.07)	0.12 (0.03)	0.15 (0.04)	0.21 (0.07)
FSAm-NPS dietary index	6.93 (1.35)	6.15 (1.16)	6.86 (1.09)	7.78 (1.26)

Values are expressed as the mean (SD) for normally distributed continuous variables or median [interquartile range] for non-normally distributed continuous variables, or n (%) for categorical variables

BMI, body mass index; HDL cholesterol, high-density lipoprotein cholesterol; LDL cholesterol, low-density lipoprotein cholesterol; T2DM, type-2 diabetes mellitus; UPF, ultra-processed foods

As expected, a higher consumption of UPF (energy ratio) was characterized by higher intakes of meat, non-alcoholic beverages, carbohydrates, fats, protein, and lower intakes of vegetables and fruits (Table 2), whereas no association was evident for UPF with cereals and fats. Stratifying dietary intake by FSAm-NPS dietary index revealed a similar distribution, where the highest FSAm-NPS dietary index tertile group had the highest consumption of meat, carbohydrates, and fats, and the lowest consumption of vegetables.

**Table 2:** Habitual dietary intake of main food groups and macronutrients across tertiles of ultra-processed foods (UPF) intake, and tertiles of Food Standards Agency nutrient profiling system (FSAm-NPS) dietary index

	Tertiles of UPF intake, energy ratio			Tertiles of FSAm-NPS dietary index			
Dietary Variable	Low (highest nutritional quality) N = 487	Medium N = 486	High N = 487	Low (highest nutritional quality) N = 487	Medium N = 486	High N = 487	
Potatoes and other tubers (g/day)	61.1 (22.4)	61.6 (22.2)	57.2 (19.8)	63.7 (23.4)	60.0 (21.1)	56.2 (19.4)	
Vegetables (g/day)	199.8 (65.2)	170.8 (53.0)	156.9 (51.1)	207.6 (64.5)	174.0 (50.8)	145.9 (44.3)	
Pulses (g/day)	7.3 (4.9)	6.0 (4.4)	5.2 (3.5)	7.3 (4.9)	6.1 (3.6)	5.0 (4.3)	
Fruits and nuts (g/day)	197.9 (94.2)	173.6 (79.9)	139.5 (75.3)	230.5 (86.5)	164.5 (72.2)	116.0 (57.9)	
Dairy products (g/day)	210.6 (107.1)	190.5 (100.0)	194.3 (110.8)	246.5 (120.1)	188.0 (92.9)	160.8 (84.1)	
Cereals and cereal products (g/day)	168.6 (50.3)	167.5 (47.8)	169.2 (45.1)	171.1 (52.5)	162.9 (46.8)	171.2 (43.2)	
Meat and meat products (g/day)	100.5 (36.2)	113.1 (38.2)	133.2 (47.8)	94.0 (29.5)	109.5 (35.9)	143.2 (46.6)	
Fish and crustaceans (g/day)	22.5 (13.9)	21.2 (14.4)	18.4 (10.9)	23.0 (15.2)	20.1 (12.4)	19.0 (11.6)	
Eggs and egg products (g/day)	18.1 (13.0)	17.5 (10.7)	15.8 (10.4)	17.6 (11.1)	17.4 (12.0)	16.3 (11.2)	
Sugars and sweets (g/day)	33.7 (12.6)	37.1 (14.8)	42.2 (16.9)	33.7 (13.2)	37.9 (14.7)	41.4 (16.7)	
Cake (g/day)	48.6 (15.8)	54.4 (18.7)	57.2 (22.7)	51.9 (17.4)	54.2 (19.8)	54.1 (21.4)	
Non-alcoholic beverages (g/day)	1,520.0 (267.8)	1,539.6 (264.1)	1,602.2 (317.3)	1,576.2 (278.0)	1,538.5 (262.7)	1,547.1 (314.2)	
Alcoholic beverages (g/day)	183.9 (212.0)	202.3 (224.5)	212.2 (233.7)	128.7 (169.8)	185.4 (204.8)	284.3 (259.0)	
Carbohydrates (g/day)	189.26 (45.18)	196.24 (47.20)	212.28 (53.28)	202.07 (49.92)	193.45 (48.70)	202.24 (49.72)	
Fats (g/day)	73.99 (15.27)	77.20 (16.06)	82.32 (18.50)	73.43 (15.42)	75.58 (16.30)	84.50 (17.21)	
Protein (g/day)	68.49 (14.40)	68.94 (14.46)	72.07 (15.33)	70.20 (14.36)	67.02 (14.78)	72.29 (14.83)	

Values are expressed as the mean (SD)

UPF, ultra-processed foods

**Fig. 1:**
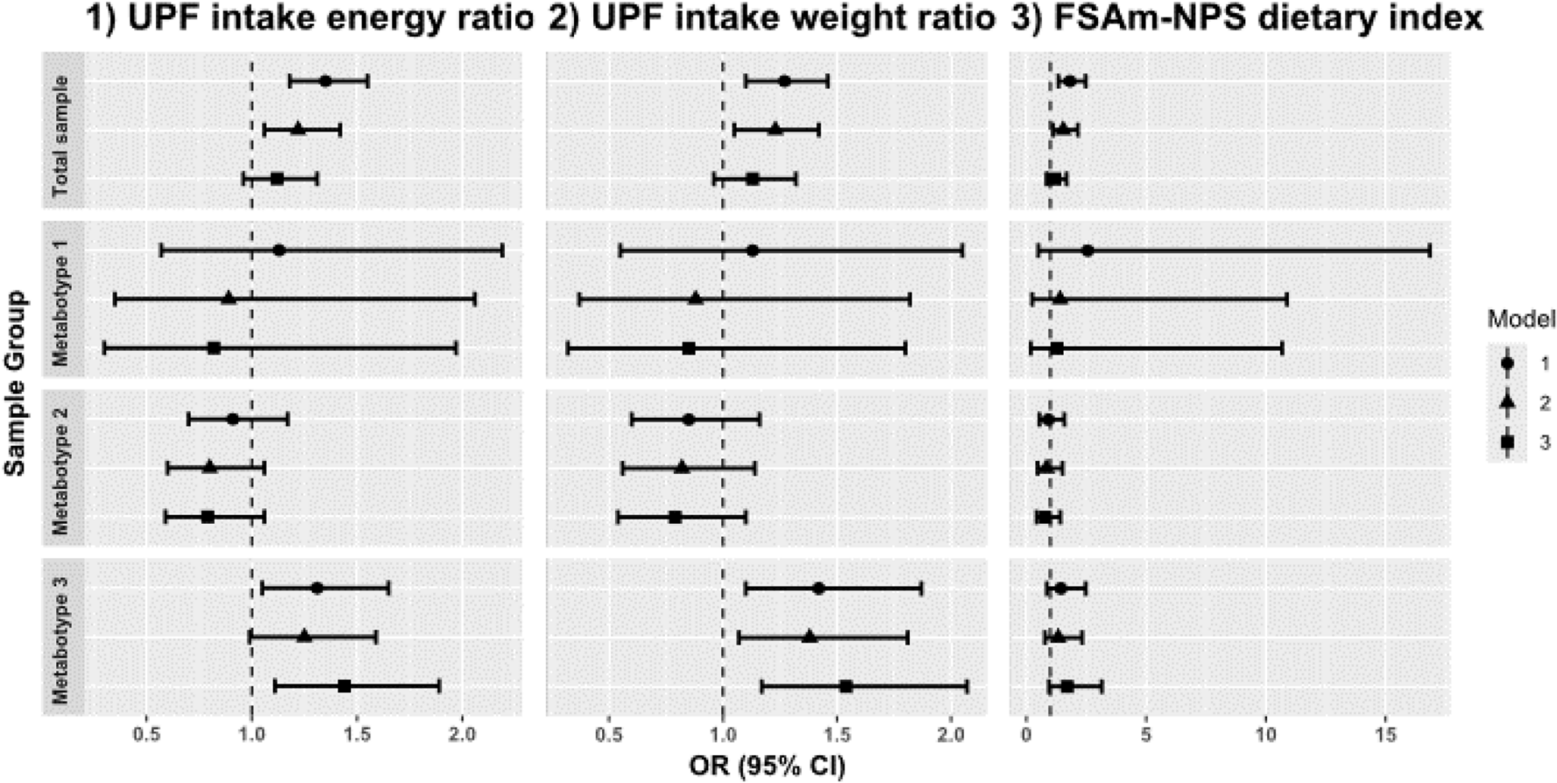
Associations between ultra-processed foods (UPF) intake and Food Standards Agency nutrient profiling system (FSAm-NPS) dietary index with type 2 diabetes (T2DM) in the total sample, and stratified by metabotype. Model 1 adjusted for age, sex, total energy intake. Model 2 additionally adjusted for education, physical activity, smoking. Model 3 additionally adjusted waist circumference and hypertension. For UPF intake (energy ratio), the variable total energy intake was not in models. The T2DM was defined as individuals with undetected or prevalent type 2 diabetes mellitus. The interaction between metabotype and dietary scores were found to be significant in all models (*p-interaction* < 0.001). UPF, ultra-processed foods; T2DM, type-2 diabetes mellitus; OR, odds ratio; CI, confidence interval. Shown are per 5% increase of UPF intake ratio and per 2-point increase of FSAm-NPS dietary index

The associations between UPF intake and T2DM were shown in Fig. 1. In the total study sample, a per 5% increase in UPF intake ratio was associated with elevated odds of T2DM for both energy [OR (95% CI): 1.35 (1.18, 1.55)] and weight ratios [OR (95% CI): 1.27 (1.10, 1.46)], following adjustment with model 1 (without the metabotype variable). On further adjustment (model 2), the significance persisted for the energy ratio [OR (95% CI): 1.22 (1.06, 1.42)], and weight ratio [OR (95% CI): 1.23 (1.05, 1.42)]. No association was detected between UPF intake and T2DM after including the metabotype for all models. However, significant interaction terms (*p*-*interaction* < 0.001) were found for metabotype and UPF intake in all models. In stratified analyses, significant associations were only seen in the third cluster. After adjustment using model 1, UPF intake [OR (95% CI) for energy ratio: 1.31 (1.05, 1.65); OR (95% CI) for weight ratio: 1.42 (1.10, 1.87)] was positively associated with T2DM. The association remained significant but decreased slightly for UPF intake weight ratio [OR (95% CI): 1.38 (1.07, 1.81)] after adjustment in model 2. Further adjustment in model 2 significantly attenuated the associations for energy ratio [OR (95% CI): 1.25 (0.99, 1.59)]. Regarding the FSAm-NPS dietary index, a significant positive relationship was observed between each 2-point increment of the index with prevalent T2DM in model 1 [OR (95% CI): 1.82 (1.34, 2.48)] and model 2 [OR (95% CI): 1.54 (1.11, 2.15)] only for the total sample (Fig. 1).

In the multinomial probit regression analysis (Table 3), significant positive associations were only found between FSAm-NPS dietary index and prediabetes in model 1 [OR (95% CI): 1.26 (1.11, 1.42)] and model 2 [OR (95% CI): 1.19 (1.04, 1.35)].

**Table 3:** Multinomial probit analysis for the associations between ultra-processed foods (UPF) intake and Food Standards Agency nutrient profiling system (FSAm-NPS) dietary index with glucose tolerance status

	Glucose tolerance status (N = 1460)		
	Normal	Prediabetes	T2DM
Cases	N = 771	N = 516	N = 173
	Reference	OR (95% CI)	OR (95% CI)
UPF intake, energy ratio^a^			
Model 1 ^b^	1	1.06 (0.99,1.13)	1.52 (0.44,5.25)
Model 2 ^c^	1	1.02 (0.95,1.08)	1.27 (0.62,2.59)
Model 3 ^d^	1	0.98 (0.91,1.05)	1.04 (0.81,1.32)
UPF intake, weight ratio			
Model 1 ^b^	1	1.06 (0.99,1.13)	1.43 (0.57,3.59)
Model 2 ^c^	1	1.04 (0.97,1.11)	1.29 (0.68,2.43)
Model 3 ^d^	1	1.01 (0.94,1.08)	1.01 (0.91,1.11)
FSAm-NPS dietary index			
Model 1 ^b^	1	**1.26 (1.11,1.42)**	2.42 (0.29,20.47)
Model 2 ^c^	1	**1.19 (1.04,1.35)**	1.73 (0.54,5.60)
Model 3 ^d^	1	1.06 (0.92,1.22)	1.26 (0.54,2.97)

^a^ For UPF intake (energy ratio), the variable total energy intake was not in models. ^b^: Adjusted for age, sex, total energy intake. ^c^: Additionally adjusted for education, physical activity, smoking. ^d^: Additionally adjusted waist circumference and hypertension

Glucose tolerance status was categorized into normal, prediabetes, and T2DM, following the American Diabetes Association criteria

T2DM, type-2 diabetes mellitus; UPF, ultra-processed foods; OR, odds ratio; CI, confidence interval

Shown are per 5% increase of UPF intake ratio and per 2-point increases of FSAm-NPS dietary index, respectively. P-values < 0.05 are shown in bold

The sensitivity analysis, wherein we further adjusted for hypertension and waist circumference (Model 3), showed no significant associations between dietary scores and T2DM, nor for prediabetes in total study sample (Table 3; Fig. 1). Additionally, we ran Model 3 with BMI substituted for waist circumference, the effect sizes and P-values remained similar (data not shown). The further inclusion of carbohydrates intake level (model 4) into the multivariable models strengthened the associations of the UPF intake with T2DM, whereas this adjustment did not significantly alter the association of the FSAm-NPS index with T2DM (Supplementary Tables S3–4).

## Discussion

Our population-based study found a significant positive association between diets with poor nutritional profiles or higher UPF intake and T2DM, as indicated by the FSAm-NPS dietary index and the NOVA classification, respectively. The interaction effects between both dietary scores and metabotype were found to be significant. Specifically, in metabotype-stratified subgroups, only metabotype 3, the most metabolically unfavorable group, showed a significant association between UPF consumption and T2DM. No significant relationship was found between the FSAm-NPS dietary index and T2DM in any metabotype subgroup. Concerning glucose tolerance status, a higher FSAm-NPS dietary index, i.e., a lower diet quality, was associated with prediabetes.

Earlier research suggested that diets high in FSAm-NPS dietary index or UPF, were associated with an elevated risk of chronic diseases, specifically diabetes, hypertension, cardiovascular disease, and cancer [44–48]. In accordance with previous studies [16, 49], our study suggests that both diet patterns were similar in that they consisted of substantially larger amounts of meat and sugar, along with reduced vegetables and fruits. Aside from the low nutritional density, UPF are often subjected to degradation of the food matrix, chemical modification of food substances, presence of food additives, loss of micronutrients, and exposure to packaging materials [8, 10]. Due to their pervasive accessibility and affordability, these high-energy-density options may not just account for the majority of consumers’ daily caloric intake [49, 50], but potentially reduce their consumption of health promoting foods such as whole grains, fruits, and vegetables [51]. This shift in dietary habits could, in turn, heighten the risk of adverse health outcomes [9, 50, 52].

In our sensitivity analysis, adjusting for waist circumference and hypertension weakened the association between both dietary scores and T2DM in total study sample. Since the association between diet quality and T2DM is likely mediated, at least partly, by these factors it is possible that model 3 was over-adjusted. Unfortunately, due to the cross-sectional design of our study, conducting a mediation analysis to examine the potential mediating effect is ill-advised. With regards to waist circumference, the analysis demonstrated unaltered results upon substituting it with BMI in Model 3. Given that waist circumference is even more strongly associated with the risk of developing cardiovascular disease and is more frequently used as an indicator of central adiposity than BMI [37], we chose waist circumference over BMI in analysis model 3.

### Association between dietary patterns and T2DM

Prior to including the metabotype variable, our study demonstrated a significant positive association between UPF intake (both weight ratio and energy ratio) and T2DM, aligning with the existing literature. The French Nutri Net-Santé cohort study demonstrated that a 10.0% increase in the consumption of UPF (in weight) escalated the risk of T2DM by 13.0% [53]. The higher intake of UPF has been shown to be positively associated with a higher risk for T2DM (hazard ratio 1.12 per 10% increment in UPF weight) in another prospective cohort study based on participants from the UK Biobank [54]. With a median 12-year follow-up, the SUN (Seguimiento Universidad de Navarra) project showed a 53% increased T2DM risk for participants in the highest tertile of UPF intake (in energy) compared to the lowest tertile [55]. Similar trends were also found in the other cohort studies in Netherlands and China [15, 56]. In an effort to bolster comparability and generalizability, we analyzed both the weight and energy ratios of UPF intake, diverging from most previous research that typically focused just on one of these aspects.

In our study, a diet characterized with a higher FSAm-NPS diet index, i.e., a nutritionally poorer diet, was significantly positively associated with diabetes. A significant association was also seen with prediabetes. Few studies have revealed the association between higher FSAm-NPS diet index (or Nutri-Score) and elevated glucose levels [16, 17], an observation that is consistent with our findings. To the best of our knowledge, the current study is the first to examine and identify the associations between FSAm-NPS diet index with both prediabetes and diabetes within a large sample. Notably, all non-diabetic subjects received an OGTT, ensuring an accurate characterization of glucose metabolism.

Our findings support and enhance previous research indicating that both dietary scores similarly impact health [57]. Of note Ferreiro et al. [58] observed that a greater proportion of UPF exists in the higher Nutri-Score categories, with the percentage escalating from 26.1% in category A to a staggering 83.7% in category E. As a practical and simple food labeling system, the Nutri-Score has already proven effectiveness in supporting informed decision-making about healthier food choices, and has been suggested by the EU to enhance customers’ diet quality [12, 13].

### Incorporating the metabotype concept

In our analysis, the relationship between UPF consumption and T2DM risk was confined to metabotype 3, the most metabolically unfavorable subgroup. Extensive studies have demonstrated the role of UPF in promoting the development of T2DM [14, 15, 53–56]; however, they largely overlooked individual factors such as genetics and metabolism, which led to a one-size-fits-all approach in dietary guidelines. Our study differentiates itself by scrutinizing the varied responses—or lack thereof—to dietary patterns across multiple metabotypes, potentially enabling more precise dietary suggestions.

Some researchers argue that the efficacy of dietary interventions can be contingent on individual-specific factors; e.g., the impact of vitamin D [59], breads [60], and red wine polyphenols [61] has been found to significantly differ among various metabotypes. This notion is further substantiated by studies utilizing the decision tree method to deliver personalized dietary recommendations for specific metabolic subgroups [21, 62]. Our research group, in its prior investigations, has also explored the relationship between diet and T2DM, identifying distinct associations only in specific metabotype clusters [22, 23]. Similar to our current results, we have also previously reported stronger associations between common dietary patterns-quantified by the Alternate Healthy Eating Index and the Mediterranean Diet Score—and T2DM in metabotype 3 compared with the total study group [22]. In addition, applying the same metabotype subgrouping approach as in this study, different reactions to the OGTT or dietary fiber supplementation across metabotypes were observed in another study population [63]. Our finding suggests a potentially more pronounced impact of UPF consumption on individuals with unhealthy metabolic profile, supporting the argument for metabotype-specific dietary recommendations over generalized dietary advice [18–20].

Subjects in metabotype cluster 3 exhibited relatively adverse demographic characteristics, which are recognized risk factors for diabetes [1, 3], and a higher prevalence of prediabetes, T2DM, and hypertension, demonstrating the metabotype concept’s effectiveness in stratifying populations into distinct metabolic subgroups [34]. To further confirm our hypothesis, as well as deepen our understanding of the intricate interplay between dietary patterns, metabotype, and diabetes risk, it is necessary to conduct future prospective studies with larger sample size. Such an approach could help formulate tailored dietary recommendations for different metabotype subgroups and implement personalized disease prevention strategies on a population-wide scale.

### Strengths and limitations

Our relatively large sample size, comprehensive dietary data collected via combined repeated 24HFLs and an FFQ, and detailed glucose tolerance status information strengthened our study. Regarding the cross-sectional study design, our findings can provide insights into potential associations and generate hypotheses on dietary recommendation, rather than establishing causality. Also, subgrouping resulted in a smaller participants number in each metabotype. The recall bias and the potential for under- or over-reporting inherent in dietary data collection cannot be entirely circumvented. Population loss during follow-up from KORA S4 to FF4 and the exclusion criteria may have introduced selection bias. Also, the definition of “metabotype” is yet to be standardized. Nevertheless, we employed an optimized metabotype as proposed by Dahal et al. [34], which reduced the previous comprehensive set of 32 [35] and 16 [23] parameters down to just 5 that are measured routinely. This approach simplifies the metabotype concept while preserving its validity, potentially facilitating its wider dissemination and application.

## Conclusion

Taken together, the study suggests that poorer diet quality or higher UPF intake, evaluated by FSAm-NPS dietary index and NOVA classification, respectively, were significantly associated with T2DM. Poorer overall dietary quality is also related to prediabetes. Notably, across different metabotype subgroups, the association between UPF consumption and T2DM was only found in subjects with the metabolically most unfavorable metabotype 3. This study emphasizes the potential benefits of personalized or stratified dietary interventions as a means to address the challenge of reducing diabetes incidence. Future studies evaluating long-term diabetes outcomes are needed before a metabotype-specific dietary recommendation can be fully endorsed.

## Electronic supplementary material

Below is the link to the electronic supplementary material.

Supplementary Material 1

## Data Availability

The data underlying this article cannot be shared publicly due to data protection reasons. The data will be shared on reasonable request to the corresponding author.

## Abbreviations

BMI: Body Mass Index
CI: Confidence Interval
FFQ: Food Frequency Questionnaire
FSAm-NPS: Modified Food Standards Agency Nutrient Profiling System
HDL cholesterol: High Density Lipoprotein cholesterol
hs C-reactive protein: High-Sensitivity C-reactive protein
KORA: Cooperative Health Research in the Region of Augsburg
LDL cholesterol: Low-density lipoprotein cholesterol
NAKO: German National Cohort
OGTT: Oral Glucose Tolerance Test
OR: Odds Ratio
T2DM: Type 2 Diabetes Mellitus
UPF: Ultra-Processed Foods
24HFL: 24-Hour Food Lists
