# Supplementary material for: Differential association of dietary scores with the risk of type 2 diabetes by metabotype
Source: Eur J Nutr. 2024 May 7;63(6):2137–48. doi: 10.1007/s00394-024-03411-0 (PMC11377363; doi:10.1007/s00394-024-03411-0)
Supplement: Supplementary file 1 — Supplementary Material 1 [file 394_2024_3411_MOESM1_ESM.doc]

**Supplemental material**

**Supplementary Table S.1.** Baseline characteristics of KORA FF4 study participants, overall and stratified by tertiles of Food Standards Agency nutrient profiling system (FSAm-NPS) dietary index.

|  |  | **Tertiles of FSAm-NPS dietary index** | | |  |
| --- | --- | --- | --- | --- | --- |
|  | **Overall** | **Low** (highest nutritional quality) | **Medium** | **High** (lowest nutritional quality) |  |
| N | 1460 | 487 | 486 | 487 |  |
| Sex, n (%) |  |  |  |  |  |
| Male | 690 (47.3) | 138 (28.3) | 214 (44.0) | 338 (69.4) |  |
| Female | 770 (52.7) | 349 (71.7) | 272 (56.0) | 149 (30.6) |  |
| Age (years) | 59.2 (11.8) | 62.3 (11.8) | 60.4 (11.3) | 54.8 (11.0) |  |
| Education, n (%) |  |  |  |  |  |
| < 10 years | 76 (5.2) | 33 (6.8) | 20 (4.1) | 23 (4.7) |  |
| 10–12 years | 837 (57.3) | 236 (48.5) | 299 (61.5) | 302 (62.0) |  |
| ≥ 13 years | 547 (37.5) | 218 (44.8) | 167 (34.4) | 162 (33.3) |  |
| Family history of diabetes, n (%) |  |  |  |  |  |
| Yes | 472 (32.3) | 173 (35.5) | 141 (29.0) | 158 (32.4) |  |
| No | 856 (58.6) | 264 (54.2) | 298 (61.3) | 294 (60.4) |  |
| Not specificized | 132 (9.0) | 50 (10.3) | 47 (9.7) | 35 (7.2) |  |
| Metabotype, n (%) |  |  |  |  |  |
| 1 | 218 (14.9) | 66 (13.6) | 71 (14.6) | 81 (16.6) |  |
| 2 | 1029 (70.5) | 370 (76.0) | 344 (70.8) | 315 (64.7) |  |
| 3 | 213 (14.6) | 51 (10.5) | 71 (14.6) | 91 (18.7) |  |
| BMI (kg/m^2^) | 27.46 (4.84) | 26.60 (4.40) | 27.46 (4.54) | 28.32 (5.36) |  |
| BMI categorized, n (%) |  |  |  |  |  |
| Underweight | 6 (0.4) | 3 (0.6) | 2 (0.4) | 1 (0.2) |  |
| Normal weight | 476 (32.6) | 197 (40.5) | 144 (29.6) | 135 (27.7) |  |
| Overweight | 611 (41.8) | 189 (38.8) | 219 (45.1) | 203 (41.7) |  |
| Obese | 367 (25.1) | 98 (20.1) | 121 (24.9) | 148 (30.4) |  |
| Waist circumference (cm) | 95.8 (14.1) | 91.8 (12.6) | 95.8 (13.3) | 99.7 (15.1) |  |
| Physical activity, n (%) |  |  |  |  |  |
| Active | 909 (62.3) | 372 (76.4) | 307 (63.2) | 230 (47.2) |  |
| Inactive | 551 (37.7) | 115 (23.6) | 179 (36.8) | 257 (52.8) |  |
| Smoking status, n (%) |  |  |  |  |  |
| Current | 204 (14.0) | 34 (7.0) | 71 (14.6) | 99 (20.3) |  |
| Former | 560 (38.4) | 184 (37.8) | 192 (39.5) | 184 (37.8) |  |
| Never | 696 (47.7) | 269 (55.2) | 223 (45.9) | 204 (41.9) |  |
| Alcohol consumption (g/day) | 7.0 [0.2, 22.9] | 2.9 [0.0, 17.1] | 7.9 [0.9, 22.9] | 11.4 [1.6, 29.2] |  |
| Fasting serum glucose (mg/dl) | 97.0 [91.0, 106.0] | 96.0 [89.5, 105.0] | 97.5 [91.0, 107.0] | 98.0 [92.0, 107.0] |  |
| hs C-reactive protein (mg/L) | 1.12 [0.57, 2.40] | 1.00 [0.54, 1.99] | 1.30 [0.64, 2.86] | 1.10 [0.53, 2.55] |  |
| Total-cholesterol (mg/dl) | 218.8 (39.5) | 218.2 (39.7) | 221.4 (40.0) | 216.8 (38.7) |  |
| HDL cholesterol (mg/dl) | 64.0 [53.0, 78.6] | 70.0 [58.0, 82.5] | 64.0 [52.8, 77.9] | 60.0 [49.2, 74.0] |  |
| non-HDL cholesterol (mg/dl) | 152.0 (40.1) | 147.2 (39.5) | 154.6 (39.9) | 154.2 (40.5) |  |
| LDL cholesterol (mg/dl) | 136.1 (35.5) | 133.7 (35.3) | 138.4 (35.6) | 136.2 (35.5) |  |
| Triglycerides (mg/dl) | 104.9 [75.7, 142.0] | 95.7 [71.6, 125.0] | 110.50 [79.0, 148.3] | 110.2 [76.8, 152.9] |  |
| Serum uric acid (mg/dl) | 5.49 [4.51, 6.63] | 5.06 [4.26, 6.21] | 5.61 [4.54, 6.68] | 5.81 [4.84, 6.89] |  |
| Hypertension, n (%) |  |  |  |  |  |
| No | 897 (61.5) | 295 (60.8) | 295 (60.7) | 307 (63.0) |  |
| Yes | 561 (38.5) | 190 (39.2) | 191 (39.3) | 180 (37.0) |  |
| Glucose tolerance status, n (%) |  |  |  |  |  |
| Normal glucose tolerance | 771 (52.8) | 274 (56.3) | 245 (50.4) | 252 (51.7) |  |
| Prediabetes | 516 (35.3) | 154 (31.6) | 190 (39.1) | 172 (35.3) |  |
| Undetected diabetes | 58 (4.0) | 18 (3.7) | 12 (2.5) | 28 (5.7) |  |
| Prevalent diabetes | 115 (7.9) | 41 (8.4) | 39 (8.0) | 35 (7.2) |  |
| T2DM, n (%) |  |  |  |  |  |
| No | 1287 (88.2) | 428 (87.9) | 435 (89.5) | 424 (87.1) |  |
| Yes | 173 (11.8) | 59 (12.1) | 51 (10.5) | 63 (12.9) |  |
| UPF intake, energy ratio | 0.38 (0.07) | 0.35 (0.05) | 0.38 (0.06) | 0.42 (0.06) |  |
| FSAm-NPS dietary index | 6.93 (1.35) | 5.46 (0.76) | 6.95 (0.32) | 8.38 (0.73) |  |
| Values are expressed as the mean (SD) for normally distributed continuous variables or median [interquartile range] for non-normally distributed continuous variables, or n (%) for categorical variables.  BMI, body mass index; HDL cholesterol, high-density lipoprotein cholesterol; LDL cholesterol, low-density lipoprotein cholesterol; T2DM, type-2 diabetes mellitus; UPF, ultra-processed foods. | | | | | |

**Supplementary Table S.2.** Baseline characteristics of KORA FF4 study participants, overall and stratified by metabotype.

|  |  | **Metabotype** | | |
| --- | --- | --- | --- | --- |
|  | **Overall** | **1**(healthiest metabotype) | **2** (intermediate metabotype | **3** (unfavorable metabotype) |
| N | 1460 | 218 | 1,029 | 213 |
| Sex, n (%) |  |  |  |  |
| Male | 690 (47.3) | 90 (41.3) | 461 (44.8) | 139 (65.3) |
| Female | 770 (52.7) | 128 (58.7) | 568 (55.2) | 74 (34.7) |
| Age (years) | 59.2 (11.8) | 55.8 (11.0) | 58.7 (11.7) | 65.0 (11.0) |
| Education, n (%) |  |  |  |  |
| < 10 years | 76 (5.2) | 9 (4.1) | 54 (5.2) | 13 (6.1) |
| 10–12 years | 837 (57.3) | 128 (58.7) | 574 (55.8) | 135 (63.4) |
| ≥ 13 years | 547 (37.5) | 81 (37.2) | 401 (39.0) | 65 (30.5) |
| Family history of diabetes, n (%) |  |  |  |  |
| Yes | 472 (32.3) | 60 (27.5) | 324 (31.5) | 88 (41.3) |
| No | 856 (58.6) | 139 (63.8) | 621 (60.3) | 96 (45.1) |
| Not specificized | 132 (9.0) | 19 (8.7) | 84 (8.2) | 29 (13.6) |
| BMI (kg/m^2^) | 27.46 (4.84) | 25.19 (3.18) | 26.73 (3.93) | 33.32 (5.78) |
| BMI categorized, n (%) |  |  |  |  |
| Underweight | 6 (0.4) | 3 (1.4) | 3 (0.3) | 0 (0.0) |
| Normal weight | 476 (32.6) | 105 (48.2) | 361 (35.1) | 10 (4.7) |
| Overweight | 611 (41.8) | 90 (41.3) | 471 (45.8) | 50 (23.5) |
| Obese | 367 (25.1) | 20 (9.2) | 194 (18.9) | 153 (71.8) |
| Waist circumference (cm) | 95.8 (14.1) | 90.1 (11.1) | 93.4 (12.2) | 112.9 (12.8) |
| Physical activity, n (%) |  |  |  |  |
| Active | 909 (62.3) | 141 (64.7) | 681 (66.2) | 87 (40.8) |
| Inactive | 551 (37.7) | 77 (35.3) | 348 (33.8) | 126 (59.2) |
| Smoking status, n (%) |  |  |  |  |
| Current | 204 (14.0) | 51 (23.4) | 139 (13.5) | 14 (6.6) |
| Former | 560 (38.4) | 64 (29.4) | 384 (37.3) | 112 (52.6) |
| Never | 696 (47.7) | 103 (47.2) | 506 (49.2) | 87 (40.8) |
| Alcohol consumption (g/day) | 7.0 [0.2, 22.9] | 2.9 [0.0, 17.1] | 7.8 [1.6, 22.9] | 8.6 [0.0, 30.3] |
| Fasting serum glucose (mg/dl) | 97.0 [91.0, 106.0] | 94.0 [88.0, 100.0] | 96.0 [90.0, 102.0] | 121.0 [110.0, 140.0] |
| hs C-reactive protein (mg/L) | 1.12 [0.57, 2.40] | 0.93 [0.44, 1.99] | 1.01 [0.53, 2.08] | 2.32 [1.23, 4.36] |
| Total-cholesterol (mg/dl) | 218.8 (39.5) | 248.9 (40.0) | 215.5 (36.2) | 203.7 (39.3) |
| HDL cholesterol (mg/dl) | 64.0 [53.0, 78.6] | 56.6 [49.0, 65.0] | 70.0 [59.0, 83.0] | 51.0 [44.0, 59.5] |
| non-HDL cholesterol (mg/dl) | 152.0 (40.1) | 191.5 (39.1) | 143.7 (35.3) | 151.7 (38.7) |
| LDL cholesterol (mg/dl) | 136.1 (35.5) | 169.4 (33.9) | 130.6 (31.8) | 128.6 (35.2) |
| Triglycerides (mg/dl) | 104.9 [75.7, 142.0] | 127.2 [93.4, 184.7] | 93.0 [69.0, 125.0] | 145.6 [109.0, 202.0] |
| Serum uric acid (mg/dl) | 5.49 [4.51, 6.63] | 4.51 [3.92, 5.48] | 5.46 [4.55, 6.44] | 7.08 [5.91, 8.04] |
| Hypertension, n (%) |  |  |  |  |
| No | 897 (61.5) | 171 (78.8) | 678 (66.0) | 48 (22.5) |
| Yes | 561 (38.5) | 46 (21.2) | 350 (34.0) | 165 (77.5) |
| Glucose tolerance status, n (%) |  |  |  |  |
| Normal glucose tolerance | 771 (52.8) | 148 (67.9) | 615 (59.8) | 8 (3.8) |
| Prediabetes | 516 (35.3) | 65 (29.8) | 363 (35.3) | 88 (41.3) |
| Undetected diabetes | 58 (4.0) | 3 (1.4) | 23 (2.2) | 32 (15.0) |
| Prevalent diabetes | 115 (7.9) | 2 (0.9) | 28 (2.7) | 85 (39.9) |
| T2DM, n (%) |  |  |  |  |
| No | 1287 (88.2) | 213 (97.7) | 978 (95.0) | 96 (45.1) |
| Yes | 173 (11.8) | 5 (2.3) | 51 (5.0) | 117 (54.9) |
| UPF intake, energy ratio | 0.38 (0.07) | 0.39 (0.07) | 0.38 (0.06) | 0.41 (0.07) |
| FSAm-NPS dietary index | 6.93 (1.35) | 7.05 (1.42) | 6.82 (1.33) | 7.32 (1.27) |
| Values are expressed as the mean (SD) for normally distributed continuous variables or median [interquartile range] for non-normally distributed continuous variables, or n (%) for categorical variables.  BMI, body mass index; HDL cholesterol, high-density lipoprotein cholesterol; LDL cholesterol, low-density lipoprotein cholesterol; T2DM, type-2 diabetes mellitus; UPF, ultra-processed foods. | | | | |

**Supplementary Table S.3.** Associations between ultra-processed foods (UPF) intake and type 2 diabetes (T2DM) in the total sample and stratified by metabotype.

|  | **T2DM** | | | | | | | | | | |
| --- | --- | --- | --- | --- | --- | --- | --- | --- | --- | --- | --- |
|  | **Total (N=1460)** | | **Total (N=1460) ^f^** | |  | **Cluster 1(N=218)** | | **Cluster 2(N=1029)** | | **Cluster 3(N=213)** | |
| **T2DM cases** | **N=173** | | **N=173** | |  | **N=5** | | **N=51** | | **N=117** | |
|  | **OR (95% CI)** | ***p* value** | **OR (95% CI)** | ***p* value** | ***p-***  ***interaction ^g^*** | **OR (95%CI)** | ***p* value** | **OR (95%CI)** | ***p* value** | **OR (95%CI)** | ***p* value** |
| **UPF intake, energy ratio ^a^** |  |  |  |  |  |  |  |  |  |  |  |
| Model 1 ^b^ | 1.35 (1.18,1.55) | **<0.001** | 1.15 (0.98,1.35) | 0.083 | **<0.001** | 1.13 (0.57,2.19) | 0.716 | 0.91 (0.70,1.17) | 0.465 | 1.31 (1.05,1.65) | **0.019** |
| Model 2 ^c^ | 1.22 (1.06,1.42) | **0.007** | 1.07 (0.90,1.26) | 0.445 | **<0.001** | 0.89 (0.35,2.06) | 0.795 | 0.80 (0.60,1.06) | 0.132 | 1.25 (0.99,1.59) | 0.068 |
| *Sensitivity Analysis* | | | | | | | | | | | |
| Model 3 ^d^ | 1.12 (0.96,1.31) | 0.154 | 1.07 (0.90,1.27) | 0.426 | **<0.001** | 0.82 (0.30,1.97) | 0.673 | 0.79 (0.59,1.06) | 0.119 | 1.44 (1.11,1.89) | **0.006** |
| Model 4 ^e^ | 1.22 (1.04,1.44) | **0.017** | 1.15 (0.96,1.38) | 0.122 | **<0.001** | 1.21 (0.39,3.70) | 0.730 | 0.83 (0.61,1.12) | 0.230 | 1.52 (1.16,2.03) | **0.004** |
| **UPF intake, weight ratio** |  |  |  |  |  |  |  |  |  |  |  |
| Model 1 ^b^ | 1.27 (1.10,1.46) | **0.001** | 1.18 (0.99,1.40) | 0.056 | **<0.001** | 1.13 (0.55,2.05) | 0.702 | 0.85 (0.60,1.16) | 0.348 | 1.42 (1.10,1.87) | **0.009** |
| Model 2 ^c^ | 1.23 (1.05,1.42) | **0.008** | 1.14 (0.95,1.35) | 0.159 | **<0.001** | 0.88 (0.37,1.82) | 0.743 | 0.82 (0.56,1.14) | 0.274 | 1.38 (1.07,1.81) | **0.016** |
| *Sensitivity Analysis* | | | | | | | | | | | |
| Model 3 ^d^ | 1.13 (0.96,1.32) | 0.124 | 1.14 (0.95,1.36) | 0.148 | **<0.001** | 0.85 (0.32,1.80) | 0.700 | 0.79 (0.54,1.10) | 0.197 | 1.54 (1.17,2.07) | **0.003** |
| Model 4 ^e^ | 1.25 (1.06,1.48) | **0.008** | 1.23 (1.02,1.49) | **0.030** | **<0.001** | 1.19 (0.39,3.23) | 0.737 | 0.90 (0.61,1.27) | 0.587 | 1.59 (1.18,2.20) | **0.003** |
| ^a^: For UPF intake (energy ratio), the variable total energy intake was not in models. ^b^: Adjusted for age, sex, total energy intake. ^c^: Additionally adjusted for education, physical activity, smoking. ^d^: Additionally adjusted waist circumference and hypertension. ^e^: Additionally adjusted for carbohydrates intake. ^f^: Further adjusted for metabotype. ^g^: Interaction between metabotype and UPF intake ratio.  The T2DM was defined as individuals with undetected or prevalent type 2 diabetes mellitus.  UPF, ultra-processed foods; T2DM, type-2 diabetes mellitus; OR, odds ratio; CI, confidence interval.  Shown are per 5% increase of UPF intake ratio. P-values< 0.05 are shown in bold. | | | | | | | | | | | |

**Supplementary Table S.4.** Associations between Food Standards Agency nutrient profiling system (FSAm-NPS) dietary index and type 2 diabetes (T2DM) in the total sample and stratified by metabotype.

|  | **T2DM** | | | | | | | | | | |
| --- | --- | --- | --- | --- | --- | --- | --- | --- | --- | --- | --- |
|  | **Total (N=1460)** | | **Total (N=1460) ^e^** | |  | **Cluster 1(N=218)** | | **Cluster 2(N=1029)** | | **Cluster 3(N=213)** | |
| **T2DM cases** | **N=173** | | **N=173** | |  | **N=5** | | **N=51** | | **N=117** | |
|  | **OR (95% CI)** | ***p* value** | **OR (95% CI)** | ***p* value** | ***p-***  ***interaction* ^f^** | **OR (95%CI)** | ***p* value** | **OR (95%CI)** | ***p* value** | **OR (95%CI)** | ***p* value** |
| **FSAm-NPS dietary index** |  |  |  |  |  |  |  |  |  |  |  |
| Model 1 ^a^ | 1.82 (1.34,2.48) | **<0.001** | 1.27 (0.89,1.82) | 0.190 | **<0.001** | 2.56 (0.50,16.85) | 0.292 | 0.92 (0.54,1.59) | 0.767 | 1.44 (0.86,2.48) | 0.176 |
| Model 2 ^b^ | 1.54 (1.11,2.15) | **0.010** | 1.13 (0.78,1.66) | 0.514 | **<0.001** | 1.41 (0.24,10.88) | 0.719 | 0.84 (0.47,1.51) | 0.550 | 1.33 (0.78,2.33) | 0.304 |
| *Sensitivity Analysis* | | | | | | | | | | | |
| Model 3 ^c^ | 1.19 (0.84,1.68) | 0.328 | 1.14 (0.78,1.67) | 0.514 | **<0.001** | 1.28 (0.18,10.68) | 0.805 | 0.78 (0.43,1.42) | 0.408 | 1.71 (0.95,3.15) | 0.079 |
| Model 4 ^d^ | 1.04 (0.73,1.50) | 0.816 | 1.04 (0.70,1.56) | 0.833 | **<0.001** | 0.97 (0.10,10.30) | 0.979 | 0.56 (0.30,1.05) | 0.069 | 1.86 (1.02,3.52) | **0.049** |
| ^a^: Adjusted for age, sex, total energy intake. ^b^: Additionally adjusted for education, physical activity, smoking. ^c^: Additionally adjusted waist circumference and hypertension. ^d^: Additionally adjusted for carbohydrates intake. ^e^: Further adjusted for metabotypes. ^f^: Interaction between metabotype and FSAm-NPS dietary index.  The T2DM was defined as individuals belonging to undetected diabetes mellitus or prevalent diabetes groups.  T2DM, type-2 diabetes mellitus; OR, odds ratio; CI, confidence interval.  Shown are per 2-point increase of the FSAm-NPS dietary index. P-values< 0.05 are shown in bold. | | | | | | | | | | | |


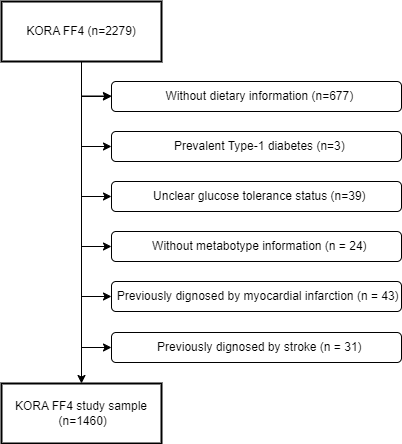


**Supplementary Fig. S.1.** Flowchart showing sample sizes and exclusions of FF4


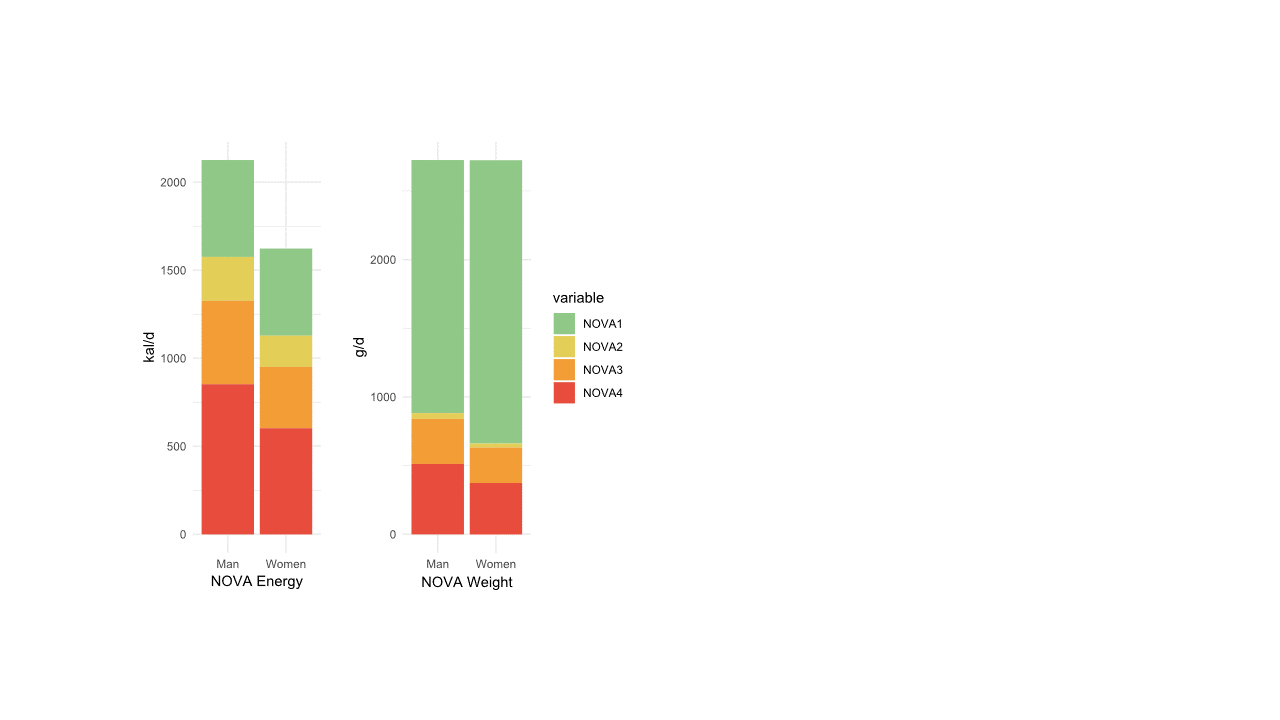


**Supplementary Fig. S.2.** Gender-stratified stacked bar showing energy and weight values of NOVA Classification groups in the dietary intake of the FF4 study population
